# Improving the Usefulness and Use of Patient Survey Programs: National Health Service Interview Study

**DOI:** 10.2196/jmir.8806

**Published:** 2018-04-24

**Authors:** Kelsey Flott, Ara Darzi, Sarah Gancarczyk, Erik Mayer

**Affiliations:** ^1^ Centre for Health Policy Imperial College London London United Kingdom; ^2^ Picker Institute Europe Oxford United Kingdom

**Keywords:** patient experience, surveys, patient data

## Abstract

**Background:**

A growing body of evidence suggests a concerning lag between collection of patient experience data and its application in service improvement. This study aims to identify what health care staff perceive to be the barriers and facilitators to using patient-reported feedback and showcase successful examples of doing so.

**Objective:**

This study aimed to apply a systems perspective to suggest policy improvements that could support efforts to use data on the frontlines.

**Methods:**

Qualitative interviews were conducted in eight National Health Service provider locations in the United Kingdom, which were selected based on National Inpatient Survey scores. Eighteen patient-experience leads were interviewed about using patient-reported feedback with relevant staff. Interviews were transcribed and underwent thematic analysis. Staff-identified barriers and facilitators to using patient experience feedback were obtained.

**Results:**

The most frequently cited barriers to using patient reported feedback pertained to interpreting results, understanding survey methodology, presentation of data in both national Care Quality Commission and contractor reports, inability to link data to other sources, and organizational structure. In terms of a wish list for improved practice, staff desired more intuitive survey methodologies, the ability to link patient experience data to other sources, and more examples of best practice in patient experience improvement. Three organizations also provided examples of how they successfully used feedback to improve care.

**Conclusions:**

Staff feedback provides a roadmap for policy makers to reconsider how data is collected and whether or not the national regulations on surveys and patient experience data are meeting the quality improvement needs of local organizations.

## Introduction

### National Patient Feedback Surveys

Involving patients in their care has become a key feature of health care improvement policies across countries and health systems [1]. The value of patient experience has been recognized not only in terms of its centrality to respectful and conscientious care, but also its relationship to better clinical outcomes and pathway adherence [2]. Feedback on experience is now collected as a norm and regarded as a fundamental quality measure [3,4]. In the United Kingdom, the National Patient Survey Program (NPSP) was established in 2002 to systematically solicit feedback from patients across many different care settings at nearly all National Health Service (NHS) organizations on a range of experience metrics [5]. As is the case in many other countries’ systems, the NPSP surveys are only one piece of the feedback puzzle that providers can use for quality improvement. Staff must interpret and integrate feedback from a multitude of sources such as bespoke surveys, online platforms, social media outlets, audits, complaints, and, in the case of the NHS, the Friends and Family Test (FFT) [6,7].

The activity around patient-reported feedback is impressive, and in many ways indicates an actual shift towards a patient-centric paradigm of care. Policy documents have endorsed the use of patient-reported feedback and articulated its benefit across the health sector [8]. However, harnessing this feedback for improvement is still challenging; growing evidence suggests that feedback is still not used to drive improvement [6,9-14]. In the United Kingdom for example, improvements on patient experience metrics have mostly come in response to large-scale national campaigns, with only modest improvements and some declines witnessed in other areas [15]. The gap between feedback collection and use not only represents a costly misuse of resources, as national surveys cost upwards of a £640,000 per survey per year, but it also raises ethical concerns around not acting on critical patient information [16,17].

The value of data for organizational quality improvement is not commensurate to the volume of data the system supplies. This misalignment is a symptom of the tension between national agendas and local needs. It is, in many ways, the consequence of national survey programs being set up to satisfy a national agenda rather than being designed with respect to local circumstances.

### The System

The underuse of data, and the substantial investment in it, calls into question the system around collecting, analyzing, and reporting patient experience feedback. The paramount actors in the system should arguably be the patients who report their experience and providers who use feedback for quality improvement. However, most of the decisions about how national patient experience feedback collections operate are made by a host of other actors.

In the NHS, the independent health care regulator known as the Care Quality Commission (CQC) is responsible for the content and roll-out of NPSP surveys. In addition, the Patient Survey Coordination Centre, housed within Picker Institute Europe, develops and deploys the surveys on behalf of the CQC. These two groups determine the questions included on surveys based on existing patient experience frameworks, with a focus on maintaining the ability to compare questions to those included on previous survey iterations [18]. Patients are consulted during the development and redevelopment of all NPSPs, but rarely invited to suggest entirely new concepts for the survey [19].

Regarding another nationally mandated feedback source, FFT, the system (namely NHS England and local commissioners) does allow for flexibility in questions, but it is more concerned with the volume of data the FFT can accumulate rather than the methodology by which it is collected. Organizations therefore have an incentive to boost response rates rather than include meaningful questions or make use of the data. The volume of data and its readily available nature should be a strength for most providers, especially when providers accompany their FFT collection with other rich free-text questions. However, this feedback has also become underutilized due to the existing necessity for most providers to sort through data manually or pay for external analysts. The real time vehicle has tremendous merit as an idea, but the system’s execution of it hinders meaningful use.

External bodies also determine sampling procedures in both the NPSP and FFT. In the NPSP, NHS providers can conduct the survey either “in house” or with an approved contractor. Both operations involve sampling based on a 24-page sampling document provided by the Coordination Centre. When a survey is administered “in house,” organizations complete all required tasks independently, which includes tasks such as the Disclosure and Barring Service checking each sampled patient to make sure they fit the criteria (ie, ensuring the patient is still alive), printing surveys, posting them, inputting data, and sending it to the Coordination Centre on time [20]. The sample for the National Inpatient Survey, the largest of the NPSP, has only recently moved from 850 to 1250; still a very low proportion of some hospitals’ inpatients, but a much larger proportion of others. Nonetheless, sampling is administratively burdensome [20]. For this reason, the CQC maintains a list of approved survey contractors (another key player in the system) who can do this work for providers. Providers purchase a survey package from contractors determining the extent of analyses and data presentation to which the provider is entitled. Not all contractors provide the same service, offer the same analysis, or engage with providers on an equal basis.

The complexity of the system pertaining to patient experience feedback is not unique to the NHS, and it is important to note that the number of actors involved does not discredit the information that patients relay in their feedback. Rather, this complex system presents considerations that providers in all health systems need to account for when interpreting data; It raises questions around how the national system of patient feedback can supply local providers with better data that could be more meaningfully translated into quality improvement information.

### Staff Perceptions

A King’s Fund report explained that gleaning information from experience data requires the same analytical capability as interpreting clinical data; however, that capability is often unavailable [21]. Staff across health systems consider patient feedback to be valuable but have neither the time nor the expertise to use it [22]. Evidence from the field of Patient Reported Outcome Measures suggests a similar pattern: data goes unused when staff cannot make sense of the data or do not fully understand how it was collected [14].

In 2007 work was conducted to understand staff attitudes towards the NPSP and their ability to use its data in the NHS. Findings from this work explicitly revealed staff’s concerns around using aggregate, organizational-level data to engage clinicians within specialties, and their difficulty navigating the statistical underpinnings of results [23]. Furthermore, this work put forth staff-driven recommendations for improvement, such as increased resources and organizational prioritization for patient experience. A full decade later, the lag in data use still exists. Many organizations have made well-defined attempts to use patient experience feedback, especially from national surveys, but have found their efforts thwarted by a series of barriers [17].

Underuse of data is unacceptable from a quality assurance perspective, as the requirement to perform analyses without proper resources risks key details being missed. It is frustrating from clinical and operational perspectives, as time and money are being invested with little return of insights to improve care. Even the National NHS Staff Survey demonstrated that only 20% of staff strongly agree that their organization acts on patients’ concerns [20]. It is demoralizing and dangerous from a patient perspective, as their input is going unheard and problems are persisting for others. Ultimately, it is ethically questionable, as patients have provided sensitive information but their feedback fails to drive change.

These concerns further expose the tensions between the data produced by the national system and the local needs within organizations. These factors compel inquiry into how data can be more usefully supplied to organizations so that it can serve as meaningful business intelligence for service improvement. It is crucial to understand how the national systems for patient feedback affect the use of data and how they can change to better support translation of feedback into insights for quality improvement.

### Aims and Objectives

Using the NHS as a case study, the aim of this research was to determine how the national system related to patient surveys can be improved so that it supports the local needs of organizations in their endeavors to use patient experience feedback. The first objective is to identify a diverse range of health care professionals responsible for using patient experience feedback and interview them about their experiences using patient experience feedback, the barriers that still prohibit using feedback for improvement, and their ideas for improving the system. The final objective is to identify and showcase successful attempts to overcome barriers and use patient experience feedback for improvement.

## Methods

### Case Study Selection

This study used a qualitative case study design to gather input from a range of organizations. Organizations were selected based on 12 metrics within the National Inpatient Survey, as it is currently the largest and most robust source of patient experience feedback in the NHS. Three organizations were selected based on demonstrated improvements on the 12 key National Inpatient scores between 2010 and 2014 (the most recent data at the time of sampling), while three others were selected based on demonstrated declines on the 12 key scores during the same time. These were then referred to as the “increased” group and “decreased” group, respectively. A final three organizations whose scores remained consistent for the same years were also selected. Organizations then put forward relevant staff for interview.

The 12 National Inpatient Survey questions were identified as most important to patients through principle component analyses. These 12 were also deemed by the Picker Institute Europe to be good indicators of whether or not organizations exhibited meaningful shifts in experience (Textbox 1). It is important to note that the sampling strategy did not account for the baseline from which those scores changed; this ensured that any organization demonstrating improvement could be included regardless of initial high or low experience scores.

Organizations that demonstrated a significant increase or decrease on any of these questions were recorded. A list was then compiled of all the organizations that were recorded to see which three providers had the most increases and decreases. Another group of organizations was also identified that remained the most constant. With regard to the organizations with consistent scores, selection consideration was given for size (to have a range of small, medium, and large acute organizations) and geography (to have a distribution of rural, urban, southern, and northern acute organizations) in order to maintain a degree of diversity, as there were many organizations that demonstrated no changes on the 12 questions. Selected organizations were contacted, and 8 of the 9 that were sampled agreed to participate; the only one to not take part was one of the *consistent* organizations.

### Interviews

Face-to-face, semi-structured interviews were conducted with as many staff as each organization elected to put forward. This was typically 1-3 staff per organization, and their most common job titles were Patient Experience Lead, Patient Experience Administrator, or Director/Deputy Director of Nursing. In total, 18 staff members were interviewed: seven from the *increased scores* group, seven from the *decreased scores* group, and four from the *consistent* group.

The topic guide covered questions such as staff responsibilities for using the patient-reported feedback, preferences for using it, current likes and dislikes regarding survey programs, and changes they would like to see made to it. Respondents were specifically asked about what changes they would like to see made to the system regarding patient experience feedback to facilitate better data use. Organizations in the *increased scores* group were asked to share their strategies for using patient reported feedback as a vehicle for shared learning. Staff from the *increased scores* group also submitted organizational information about how they had used patient-reported feedback in action planning and improvement.

**Questions used for grouping and sampling organizations.**

Do you feel you got enough emotional support from hospital staff during your stay?

When you had important questions to ask a doctor, did you get the answers that you could understand?

Overall did you feel you were treated with respect and dignity while you were in the hospital?

Do you think the hospital staff did everything they could to control your pain?

Did you have confidence and trust in the doctors treating you?

Did you find someone on the hospital staff to talk to about your worries and fears?

Did a member of staff tell you about medication side effects to watch for when you went home?

Were you involved as much as you wanted to be in decisions about your care and treatment?

In your opinion, how clean was the hospital room or ward you were in?

Did doctors talk in front of you as if you weren't there?

In your opinion were there enough nurses on duty to care for you in hospital?

Sometimes in a hospital, a member of staff will say one thing and another will say something quite different. Did this happen to you?

### Thematic Analysis

Interviews were recorded and transcribed if the participants gave explicit permission in their consent form. Two interviewees consented to an interview but declined to be recorded. In these two cases, notes were taken by a team member and used in place of a full transcript.

Transcripts were then uploaded into the qualitative analysis software NVivo (QRS International Pty Ltd). A thematic analysis was conducted to demarcate different themes or topics within the transcripts. This study used thematic analysis to identify information relevant to the experience of using patient experience survey data to generate improvements.

The codes were developed a priori for the most part, as they were taken from the background literature regarding possible barriers and facilitators to data use. Some codes were identified a posteriori as they occurred unexpectedly but were important to answering the research question. Specifically, the coding looked for mention of themes relevant to answering the research question and then subthemes mentioned in relation to the primary themes. Sentiment was coded to capture how respondents felt about any particular theme, particularly whether staff referenced subthemes negatively (as a barrier to data use), positively (as a facilitator to data use), or as a desire for change in patient survey data (staff wish list).

## Results

### Key Themes

Four primary themes were identified with a range of subthemes relating to each of them. The subthemes were expressed with different sentiments, which fell into three distinct categories: negative (barriers to using data), positive (facilitators to using data), and desire for change (staff wish list). The themes, subthemes, and sentiments are mapped below.

While staff were specifically probed about their ability to use NPSP data, transcripts of the conversations naturally exposed the types of data staff found most useful. Transcripts also revealed the variation in sentiments towards themes and subthemes. For example, staff would reference a particular theme (ie, survey data) and subtheme (ie, the inability to link data) as a barrier, and then that same subtheme (ie, the ability to link data) as a facilitator. The sentiment behind each theme was coded to categories’ subthemes. The four primary themes identified in interview transcripts related to survey methodologies, survey reports, survey data and organization, and staff factors that impact the ability to use patient experience data.

### Survey Methodologies

While discussing how they used patient experience survey data, one of the most common topics that staff mentioned was the survey methodology used in NPSP surveys. It was clear from staff that difficulty interpreting results, and lack of clarity around the reasons for certain methods, created barriers to using the data. Staff were concerned that the methods not only led to confusing results but were also inappropriate given the size of their organizations, as illustrated by two quotations below. Staff mentioned that in order to facilitate data use, methods should be more intuitive to staff who do not have survey training and should also include the scope for larger sample sizes. Some staff also expressed a desire for more real-time and qualitative methods to accompany NPSP results.

The other problem with the national survey is the way that they actually design it; the CQC part of it makes it really difficult.

So in a Trust that sees 1.6 million patients a year, although the majority are out-patients, 850 is a tiny sample. I know it’s increased this year but it’s still quite small

[The free text is] so much better because what it does is it elicits the things which matter to people, not what we think matter to them.

### Survey Reports

The second primary theme related to survey reports and how data is presented back to staff. In terms of data from the NPSP surveys, reports from the CQC and survey contractors are the principle source of patient survey data provided to NHS organizations. Staff felt that these reports often caused confusion. Despite many staff referencing these reports as barriers, other staff members gave examples of contractor reports facilitating the use of feedback. This finding related mostly to contractors’ ability to provide a report with more personalized information for each organization than that available in CQC reports. Staff also cited contractors’ reports being accompanied by workshops to explain the results. In terms of how staff discussed this themes in relation to their wish list of changes, the main desire expressed was for enhanced opportunity to share success stories, rather than simply receiving benchmarking tables.

So we got amber on every single question. Every single question we got the same as everybody else which just happens to be the same score that Morecombe Bay got who are in special measures...

So both having those stories and the information but also make the workshops not just around the outcome and the talks but actually the best practice workshops, maybe on a regular basis, so someone from Newcastle getting up and presenting to all the other Trusts who want to be there about discharge, next it will be Birmingham about food or whatever it might be but Picker being almost a coordinating body for that because that’s the vehicle with which it’s been done. Something like that would be good.

### Survey Data

The third primary theme identified related to the actual survey data that staff received from NPSP surveys. Most of the conversation about this topic related to the ability to link data to other quality indicators. Staff found NPSP data difficult to work with because it could not be compared at a granular level to other data sources and left them with an inexact picture of how patient experience data fits in with other organizational data. Another prominent subtheme revolved around the inability to glean which NPSP data points matter most to patients. Staff expressed an interest in more explanation of the data, support to analyze it, and better indication of what was most important from a patient perspective.

This could be related to any survey, but the idea of linking results at the patient level would help the patient know they were listened to see feedback on incident reporting to support the need for response to feedback.

What I would want is that to be linked in with complaints, so I’d love to have some kind of dashboardy thing that pulls all that stuff together.

In terms of understanding the data, I think when they come and do workshops with us or present the data we need—that’s very helpful but I need—we need them, in there, telling the story of how they collected the data and how it’s reliable.

For example, the question about the call button may mean different things to different patients, and they need to know what to improve.

### Organization and Staff Factors

The final theme identified related to the factors outside of survey programs that impacted how staff could use NPSP data. The subthemes related to aspects of organizational structure, the extent of training staff had in using survey data, and the priority given to patient experience within organizations. Some staff members mentioned that there was sometimes lack of clarity about whose responsibility it was to use patient experience data, and more frequently, the people in charge of using data did not feel sufficiently trained to do so.

Very few staff members were concerned about the priority given to patient experience in their organization; however, some did cite it as a key factor in being able to pursue improvements. This idea led to many staff members desiring more information about what other organizations had done to achieve success in patient experience.

And I think sharing that nationally, because I want to know what other people are doing, because even if it’s things that we’re doing well but we could do better, I don’t want to reinvent the wheel.

What I’d be interested in, is sharing best practice and stories from others.

**Figure 1:**
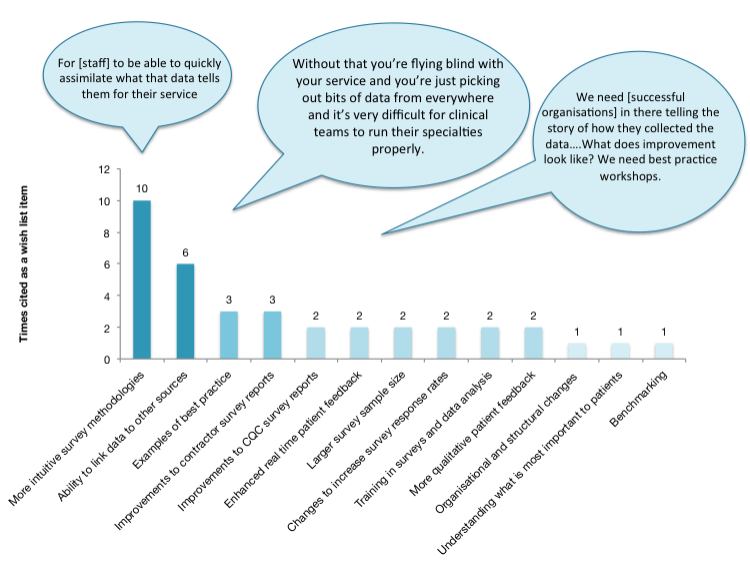
Staff wish list.

Figure 1 depicts what staff specifically said they would put on their wish list of changes in order to improve how patient-reported feedback is collected, analyzed, and presented. Examples of best practice was its own theme, but the idea of learning from others came through in a few of the themes. Staff articulated a desire for survey methodologies and resulting data to be presented in a more intuitive way that resonates with their day-to-day practice.

**Figure 2:**
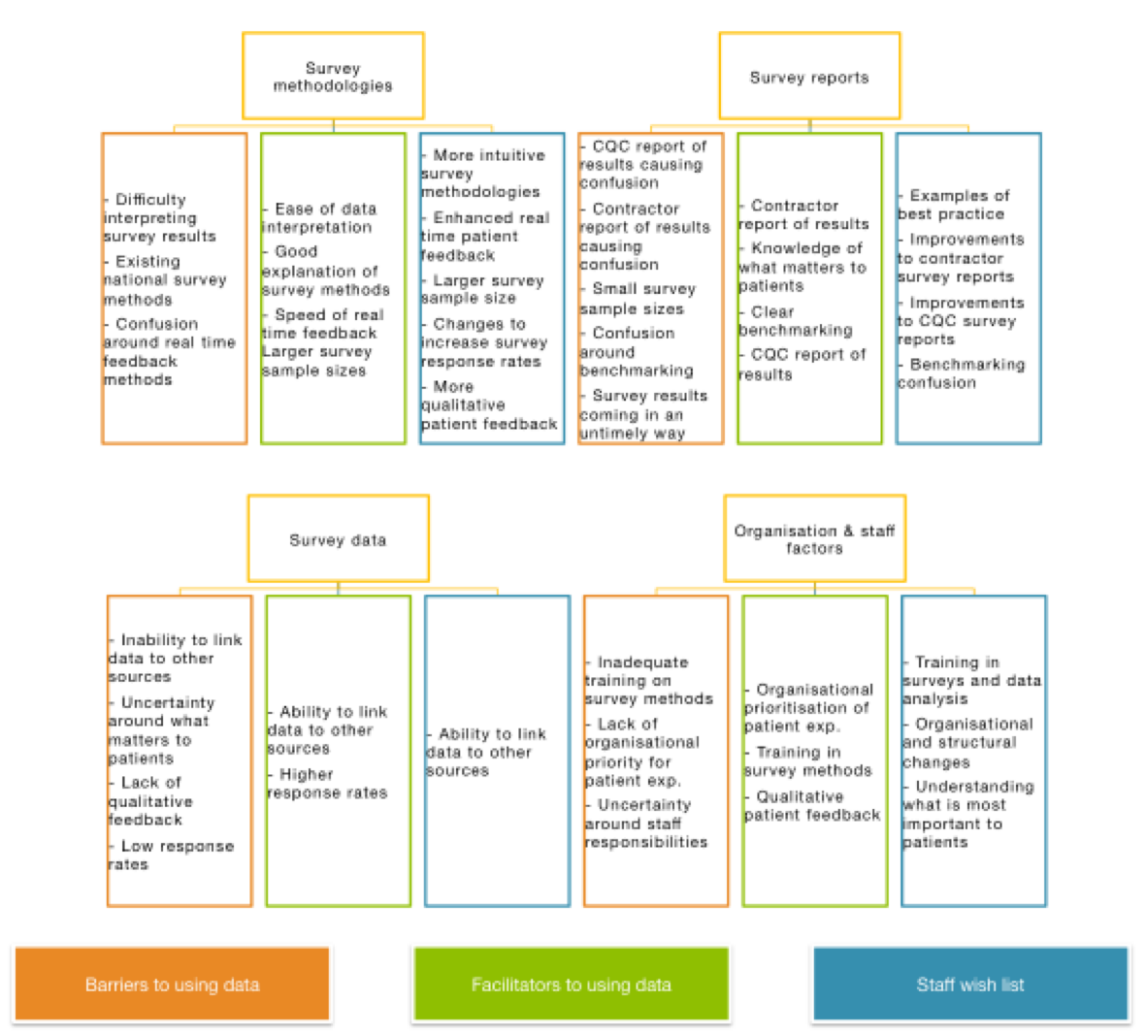
Map of themes, subthemes, and sentiment.

Finally, Textbox 2 lists the examples of how organizations in the *improved scores* category overcame identified barriers and used patient-reported feedback effectively to improve their service and their survey scores. This list reveals the general technique used and Multimedia Appendix 1 provides a full description of that process. These themes are mapped in Figure 2.

**Examples of improvements techniques.**

Data triangulation even when data cannot be directly linked

Emotional intelligence training

Identifying communication breakdowns

Bespoke survey methodologies across services

Following-up with people after they give feedback

Provision of better information about patient pathway

Values-based improvement

Competition to drive innovations

Comfort packs on the wards

Including patient experience in staff inductions

Giving staff the positive feedback from patients

Identifying priority questions on surveys

## Discussion

### Overview of Results

In total, 18 staff members who work with patient experience feedback were identified through the sampling process based on National Inpatient Survey scores; this group was deliberately diverse, representing three organizations that had demonstrated improvements, decreased performance, and consistencies in important National Inpatients Survey questions. Respondents were also from a range of small, medium, large, and teaching hospitals from across England.

The most frequently cited barriers to using patient-reported feedback had to do with interpreting results (14 mentions), understanding survey methodology (14 mentions), presentation of data in both national CQC (13 mentions) and contractor reports (12 mentions), inability to link data to other sources (7 mentions), and organizational structure (7 mentions). The most frequently cited facilitators were: ability to link data (9 mentions), ease of survey interpretation, and clarity around methodologies (7 mentions). In terms of a wish list for improved practice, staff desired more intuitive survey methodologies (10 mentions), ability to link patient experience data to other sources (6 mentions), and more examples of best practice in patient experience improvement (3 mentions).

Staff feedback varied slightly when segmented by organizational group. Organizations whose scores had decreased cited training, organizational structure, and interpreting results as barriers more often than other organizations. Those whose scores had increased focused more on difficulty understanding survey methods and confusion around CQC reports, but cited knowledge of what is most important to patients as a key facilitator. The difference in focus could relate to other aspects of organizational health. For instance, organizations with declines who cited structural issues might have obstructions to the use of patient feedback that are not necessarily a product of the system regarding patient experience feedback.

### Interpretation of Results

One of the most interesting findings emerging from the thematic analysis relates to what was *not* discussed. Even when specifically probed, staff virtually never cited finances or lack of senior-level interest as a major barrier to using patient-reported feedback. While some staff members did say they would like things like “free extra analysis,” and many cited paid-for survey contractors as providing the most useful analytic tools and workshops, staff did not feel that lack of funding was a chronic barrier to using data. Furthermore, very few staff criticized organizational leadership when citing the barriers to using data. These findings demonstrate enormous progress in the field of patient experience, as only a decade ago such feedback was highly underprioritized by staff and within budgets [23].

In terms of wish list items, the idea that received the most consensus by far, and was articulated in a variety of different themes, was the idea of sharing best practice to help other organizations emulate the successful use of data. This finding was a compelling plea for collaboration, rather than competition, to improve experiences for patients. Ideally some of the examples of improvement provided in this research will resonate with staff and provide a first step towards such shared learning. Furthermore, organizations wanted to see a variety of changes to survey methods (ie, shortening the questionnaire, offering it in different modalities, larger sampling), the ability to link data to other sources of information, clarity about how to interpret results, and better reports of results both from their contractors and the CQC. Less frequently mentioned but also on the list, organizations wanted help to improve response rates as well as changes to the analysis methods so that they include qualitative sentiment analysis.

The results also brought forward ideas for improvement strategies, such as mapping organizational values to questions, triangulating data from multiple sources to identify trends even when data is not directly linkable, sharing feedback with staff, and using “improvement maps” to gauge which questions are most important to patients (currently only provided by hired survey contractors). The underlying theme of the improvements was that chasing individual questions was not as fruitful as rectifying the root causes that are behind negative scores. This approach included a focus on integrating survey findings into conversations involving operational development to stimulate better patient experiences. More successful organizations found certain survey questions symptomatic of larger organizational health issues and recognized that improvement was going to take a more concerted effort than a single focus on one particular question. These approaches emphasized working on organizational values and staff experience rather than targeting a specific question. Furthermore, these examples support the idea that provision of clear information and supplies to make ward life more enjoyable can improve experiences without making drastic changes to care delivery. Finally, there was support for involving staff in the process of learning from feedback, both in giving them positive feedback from patients and working with them to design experience feedback collections specific to their patients.

### Limitations

The research approach identified what providers are struggling with when it comes to using patient-reported feedback. Although the case studies were relatively diverse, it would have been beneficial to gather feedback from a wider range of respondents. Furthermore, some interviewees declined to be recorded, meaning that their files were based on notes, which contained less precise information for coding. Finally, some of the results regarding successful approaches to data use cannot be taken at face value, because things like improvement maps (where certain survey questions are mapped to an overall question) are conceptually questionable in terms of providing accurate levels or importance [6].

### Implications for Health Policy

Through this research, health care staff have provided a blueprint for optimizing national systems related to patient experience feedback including how it is collected, analyzed, and presented. In order for patient-reported feedback to be an effective improvement tool, and avoid the ethical grey zone around soliciting patient input and not acting on it, feedback programs need to make efforts to facilitate data comprehension and use.

Staff have offered a considerable amount of insight into how best to improve the system regarding patient experience feedback, such that it generates useful intelligence for organizational improvement. Some of the staff’s suggestions could be seen as simple adjustments to existing surveys, such as larger sample sizes, reports that are more appropriately pitched to the audience, and revisions in survey methodologies such that they make sense to service providers. Facilitating local data use also requires the system regarding patient surveys to provide relevant data breakdowns and intuitive reports and presentations. This is true in the NHS, but also in international contexts. Not only are staff eager to have ward- and service-level data, they need survey results to explain which aspects of experience are most important to various patient groups. This approach likely requires soliciting and relaying a different kind of data entirely. Different types of data have different utilities for staff, but the feedback of staff in this study present a desire to further explore near-real-time feedback (that does not risk confidentiality) and extraction from unstructured data; more appropriately called *patient stories*.

These more difficult challenges are perhaps the most important. The idea of linking feedback to other information represents staff’s inclination to move towards more holistic quality improvement rather than continue to analyze and respond to a wide range of disparate, uninterpretable data. Enabling wish list items like this would require a paradigm shift in patient experience feedback collection.

The paradigm regarding patient experience feedback is heavily rooted in large national initiatives, the NPSP and the FFT, both of which are accompanied by a sluggish bureaucracy and political concerns. It is likely that these initiatives are neither capturing, nor producing, what is most useful to the organizations trying to use patient feedback to improve care. Listening to what staff said in this interview study should ignite a change in thinking and compel the actors within the system to collect clear, linkable, digitally mature, and timely information. Furthermore, truly understanding what matters to patients (another wish list item) requires a different level of engagement with patients beyond testing surveys and asking people what they expect from their care pathway.

These ideas for change do not suggest abolishing national survey initiatives. Currently, these initiatives still hold the only academically robust source of patient experience feedback and are likely to play a role for a long time. Rather, these ideas call for improvement in the investment in feedback collection; they demand modernizing feedback collection and revamping it to be flexible to patient priorities, which is reflective of the whole patient population and more useful to the frontline. If the system is open to new approaches, these changes will help transform unused data into business intelligence insights for clinical informatics.

### Conclusion

Experience has joined effectiveness and safety to form the quality pyramid that has been accepted by policy makers, providers, and patients. Patient-reported feedback programs are now a staple of developed health care systems; however, they have not yet achieved their full potential as a conduit for patient needs and preferences to enter into quality improvement strategies. This research demonstrates which barriers lie behind the problem. More importantly it illuminates what staff want and need from the system related to patient experience feedback, in order to put the data to use.

The focus on enhanced data presentation came through very strongly, as did the desire for patient-reported feedback to be explained in a way that is meaningful not only to analysts, but also to frontline staff. It is also clear that more needs to be done to enable data linkage so that staff can explore problems within specialties and across datasets. Finally, the most powerful finding of this study was one for a shared network of success and shared learning. The examples in this research makes inroads towards such shared learning and will hopefully be the beginning of a growing repository of successful approaches to using patient feedback that can help the system adapt to changing local needs.

## Appendix

Multimedia Appendix 1 Examples of improvement.

## Abbreviations

CQC: Care Quality Commission
FFT: Friends and Family Test
NHS: National Health Service
NIHR: National Institute for Health Research
NPSP: National Patient Survey Program
