# Supplementary material for: Improving the Usefulness and Use of Patient Survey Programs: National Health Service Interview Study
Source: J Med Internet Res. 2018 Apr 24;20(4):e141. doi: 10.2196/jmir.8806 (PMC5941087; doi:10.2196/jmir.8806)
Supplement: Multimedia Appendix 1 [file jmir_v20i4e141_app1.pdf]

Multimedia Appendix 1. Examples of improvement.

Examples of Improvement

***The worry is that sometimes the tried and tested stuff gets you the results. What we're after isn't necessarily the results; we're after that kind of world-class patient experience, which means that you've got to kind of do stuff that's different.* - Deputy Director of Patient Experience at RAL**

Improvement Technique	Improvement strategy
**Data triangulation**	We will look at [the data] and if there's any concerns to start with... then my job will be to really cross-reference it with any of the other data that we have in the organisation. So if ...some statistically significant change has happened, then I'll be looking and saying, "Right do we have any complaints or compliments around this area? Do we have any friends and family test data about it? Is this actually an anomaly or, you know, is something that we're seeing across the board?" I drill all [the data] down and then to make sense of it for the teams.
**Emotional intelligence training**	If you can improve emotional intelligence across an organisation you'll get all those all other things rights. [For example] doctors talking as if I'm not there, that's emotional intelligence. That's emotional intelligence because that's about looking after each other and making sure we're all talking from the plan and that we communicate with each other, so it's all about communication skills. Now I am constantly delivering this training across the trust.
**Identifying communication breakdowns**	I map complaints to the emotions of the complaint... the staff experience and how they were feeling emotionally.... I map the points in the patient journey of the complaint and it's usually about communication. 50% of complaints in the [organisation] are about communication. That's all that soft skills stuff and unfortunately in

	general acute trusts they have less of the soft skills stuff because it tends to be quite task orientated
**Bespoke methodologies across services**	I do bring a bit of a difference to the way they've done patient experience work here in the past... so now I get a lot of people approaching me. Basically anybody that wants to do patient experience surveys within their department will come to me first and we'll talk about what their needs are and what the best way of capturing that data is because it's not always about a survey. You could do observations of care, you could be doing, you know, all sorts of different ways... I've got probably about 45 to 50 departments working with me on those.
**Feedback follow up**	A&E really use the friends and family test. [They] got consent from patients to contact them if there was negative feedback and discussed their experiences with them and made services improvements as a result...That had an impact on the CQC national survey and you saw that we were the best in the country [on certain questions] in our A&E departments, so we were delighted with that.
**Provision of better information**	We made "Welcome Packs" which had lots of information including details about how to complain, ward routines, when the ward rounds were and how the staff will help them manage their pain, ear plugs, eye masks, socks to prevent falls, paper and pens. [Pens were] because often patients would think of a question to ask doctors but then not remember it by the time the ward round happened ... We saw immediate climb in results around [related] questions.
**Values based improvement**	So we've got our values and what we try and do now is base the improvements around the values in the strong belief that if they are the right values you will see a shift in our experience scores. So rather than chasing the score, we chase everything to do with the patients and the values and underpin the values. So if we're visibly reassuring, which is one of our values, then all of those questions

abound confidence and trust should go up. So rather than trying to chase, "Did you trust your doctor? “Did you have an action plan?" we’re saying, "Are we visibly reassuring and why are we visibly reassuring?" as kind of an example of one of the values and that’s the approach that we’ve now shifted much more towards because we think, as I say, we contract the values and we can start delivering against those values, we should see some shift.

Competition for innovations

One of the things we’re developing at the moment is a competition for staff and teams so the idea is that wards and teams, doesn’t have to be clinical, come up with a patient experience improvement idea at very high-level. We’ve then got some… respected businessmen locally that have said that they’d like to be involved with helping so they’ll go along and talk to the ward and help develop that into much more of a plan. We’ll then have a presentation…where the top two or three come and do a bit of a presentation to some people and then the one that comes out top, we’ll give up to £10,000 to pay for that improvement idea and automatically it’s going into next year’s quality account.

Packs for better ward comfort

So we’ve come up with, and we’re just about to pilot it, something from America. It’s a quiet pack and it’s a little pack that we give to patients- it’s a bit like you get on an aeroplane. It’s got an eye mask in it; it’s got a lip balm in it; it’s got Sudoku in it; it’s got a crossword in it; it’s got a pencil in it and some paper. On the basis of actually at home if you can’t sleep and you pick up a book and read it, you soon fall asleep. Actually, if you try and do something you soon fall asleep. If you are worried about something, we always say to people, if you are worried write it down but actually we don’t give people in hospital pens and paper to write it down so at home we’d say that if you can’t sleep write it down and park it until morning.

**Including experience in inductions**	To try and get staff engaged in actually how do you drive forward experience it's really quite difficult. Here, you know, people have come here as a member of staff expecting to work at a world-class place as well so you've got much more ability to say to staff, you know, "We expect you to deliver a word-class…" and what we do here, when staff join, is quite a lot of work around what the values mean but also what they're joining. So it's the only place I've joined that as part of the induction you've had to sit through an hour of the history of the trust.
**Giving staff the positive feedback from patients**	Not only have we linked [qualitative feedback] to the Wow awards so staff get recognition when they're delivering a good job but we've linked it to the NICE quality standards, to the CQC so we code it all. We also have managed to do text to phone, so we have patient voices coming in. They give consent for us to share [their feedback], so we can email them round the trust. We can use it so when people walk into induction they hear the patient voices, you know, responding to the Friends and Family Test so you can then link it.
**Identifying priority questions**	What we've done is we've recently rewritten our performance report around patient experience and we've dropped an awful lot of metrics and there's about ten questions I think- there's about ten killer questions that if we nail all of those ten that will make sure that all of our values have been worked upon and if those ten questions are good, our values must be good and therefore everything else must fall into place. So rather than chasing everything, we decide if we get those right, our values must be right and if our values are right then we must improve otherwise our values are wrong.
